# Complete Genome of *Lactococcus lactis* subsp. *cremoris* UC509.9, Host for a Model Lactococcal P335 Bacteriophage

**DOI:** 10.1128/genomeA.00119-12

**Published:** 2013-01-31

**Authors:** Stuart Ainsworth, Aldert Zomer, Victor de Jager, Francesca Bottacini, Sacha A. F. T. van Hijum, Jennifer Mahony, Douwe van Sinderen

**Affiliations:** a Department of Microbiology, University College Cork, Cork, Ireland; b Centre for Molecular and Biomolecular Informatics, Nijmegen Centre for Molecular Life Sciences, Radboud University, Medical Centre, Nijmegen, the Netherlands; c Laboratory of Microbiology, Wageningen University, Wageningen, the Netherlands; d Netherlands Bioinformatics Centre, Nijmegen, The Netherlands; e NIZO food research, Ede, The Netherlands; f Alimentary Pharmabiotic Centre, Biosciences Institute, University College Cork, Cork, Ireland

## Abstract

Here, we report the complete genome of *Lactococcus lactis* subsp. *cremoris* UC509.9, an Irish dairy starter. The circular chromosome of *L. lactis* UC509.9 represents the smallest among those of the sequenced lactococcal strains, while its large complement of eight plasmids appears to be a reflection of its adaptation to the dairy environment.

## GENOME ANNOUNCEMENT

*Lactococcus lactis* strains are used extensively worldwide for the production of fermented dairy products. Bacteriophage (phage) attack during this fermentation process can lead to slow or failed fermentations and is therefore of major economic concern (1). *L. lactis* subsp. *cremoris* UC509 is an Irish cheddar starter strain and is the lysogenic host of the model P335-type phage Tuc2009 (2–6). *L. lactis* UC509.9, whose genome sequence is presented here, is a prophage-cured Tuc2009-sensitive derivative of UC509 (7).

While lactococcal phages are subject to intensive scientific scrutiny, the specific interactions with their hosts are poorly understood. To further our understanding regarding the molecular interplay between Tuc2009 and its host, we sequenced the genome of *L. lactis* UC509.9. Sequencing was performed by Agencourt Bioscience (Beverly, MA) and Macrogen (Seoul, Republic of Korea) using a combination of 454 sequencing of a 3-kb fragment library using Roche standard procedures and of Sanger sequencing of a 36-kb insert library followed by homopolymer tract correction using Illumina sequencing. Initial sequence assembly was performed using GSassembler (Roche). Gap closure and quality improvements were performed by Sanger sequencing of gap-closing PCR products as suggested by Projector 2 (8) with the Staden package (9). Homopolymer tract single nucleotide polymorphisms (SNPs) were detected and corrected using Robust Variant detection (ROVAR) (V. de Jager, B. Renckens, R. J. Siezen, and S. A. F. T. van Hijum, unpublished data [https://trac.nbic.nl/rovar/]) applied to Illumina sequencing data as described previously (10), resulting in a >200-fold coverage of the genome. Putative protein-encoding genes were identified using Prodigal version 2.0 (11). The results were inspected using Artemis (12), with manual checking and editing using BLASTP, Pfam (13), Kyoto Encyclopedia of Genes and Genomes (KEGG) (14), and Clusters of Orthologous Groups (COG) databases (15).

The complete genome of *L. lactis* UC509.9 consists of a single circular chromosome of 2,250,427 bp (35.88% G+C content) plus eight plasmids: pCIS1 (4,263 bp), pCIS2 (5,961 bp), pCIS3 (6,159 bp), pCIS4 (7,045 bp), pCIS5 (11,676 bp), pCIS6 (40,285 bp), pCIS7 (53,051 bp), and pCIS8 (80,592 bp). The *L. lactis* UC509.9 genome is predicted to contain 2,066 protein-encoding genes, of which 168 are pseudogenes. Forty-three of these 168 pseudogenes are identical to those found in *L. lactis* subsp. *cremoris* SK11 (GenBank accession no. CP000425.1). The genome of *L. lactis* UC509.9 contains 104 transposase-encoding genes involving a total of 106,746 bp, including 42 copies of IS*182* and 29 copies of IS*981*. The combination of the smallest lactococcal chromosome identified so far and the high number of transposons and pseudogenes suggests that the genome has undergone significant genome decay while adapting to the nutrient-rich dairy environment. A region of approximately 11 kb in size not present in other *L. lactis* genomes appears to be an integrated plasmid that includes the restriction-modification system ScrFII (16). The *L. lactis* UC509.9 plasmid complement encodes various traits for adaptation to the dairy environment, such as lactose and casein metabolism.

### Nucleotide sequence accession numbers. 

The complete chromosome and plasmid complement of *L. lactis* subsp. *cremoris* UC509.9 were deposited in GenBank under accession no. CP003157 (chromosome), CP003165 (pCIS1), CP003164 (pCIS2), CP003163 (pCIS3), CP003162 (pCIS4), CP003161 (pCIS5), CP003160 (pCIS6), CP003159 (pCIS7), and CP003158 (pCIS8).
